# The impact of a perioperative pulmonary care bundle implementation on postoperative outcomes in 1,665 surgical cancer patients: experience from a tertiary referral cancer center in Jordan

**DOI:** 10.1186/s13037-020-00277-z

**Published:** 2021-01-06

**Authors:** Riad Abdel Jalil, Hani Al-Najjar, Mohamad K. Abou Chaar, Mahmoud Al-Masri, Faiez Daoud, Ali Al-Ebous, Ali Dabous, Ahmad M. Shehadeh, Samer Abdel Al, Fade Alawneh, Obada Al-Qudah, Mohammad Al-Kharabsheh, Ghazi Al-Odat, Iqbal Mohammad, Najah Hussein, Zeinab Hudaip, Asma Al-Tbakhi, Flsteen Aqel

**Affiliations:** 1 Department of Surgery, King Hussein Cancer Center, Amman, Jordan; 2 Thoracic Surgery Unit, Lung Service Tumor Board, Surgical Residency Program, King Hussein Cancer Center, Amman, 1194 Jordan; 3 Department of Nursing, King Hussein Cancer Center, Amman, Jordan; 4 Respiratory Therapy Unit, King Hussein Cancer Center Amman, Amman, Jordan

**Keywords:** Postoperative Pulmonary Complications, Perioperative Pulmonary Care Bundle, Major Cancer Surgeries

## Abstract

**Background:**

Postoperative pulmonary complications can be a major catastrophic consequence of major surgeries and can lead to increased morbidity, mortality, hospital stay, and cost. Many protocols have been tried to reduce serious adverse outcomes with effective strategies including a bundle of preoperative, intraoperative and postoperative techniques. Using these techniques maybe challenging in developing countries with limited resources even in specialized centers.

**Methods:**

A before-and-after trial comparing our data from the national surgical quality improvement program (NSQIP) based on their reports. Data was collected prospectively for the patients who underwent major surgeries at King Hussein Cancer Center during the year 2017 when the use of the perioperative pulmonary care bundle was mandatory to all surgery teams and compared it with the data collected retrospectively for the patients who underwent the same type of surgeries in the year 2016 when the use of such a bundle was optional. The primary end point is the decrease in incidence of postoperative pulmonary complications. Simple descriptive statistical analysis was used to obtain results.

**Results:**

The potential risk factors for postoperative pulmonary complications for 1665 patients divided into 2 groups (2016 vs. 2017); 764 (45.9%) vs. 901 (54.1%), respectively. There were no significant differences regarding gender (male 46.7% vs. 46.4%, *P* value = 0.891, female 53.3% vs. 53.6%, *P* value = 0.39), mean of age (53.5 year vs. 5.28 year, *P* value = 0.296), mean of body mass index (BMI) (28.6 vs. 6%, 28.6, *P* value = 0.95), smoking status; (69.6% vs. 65.1%, *P* value = 0.052), ventilator use (0.3% vs. 0.4% *P* value = 0.693) and chronic obstructive pulmonary disease (1.4% vs. 1.4 with, *P* value = 0.996).The data showed a significant reduction in postoperative pneumonia between the 2 groups (2016 vs. 2017) (2.7% vs. 0.9%, *P* value = 0.004) and showed a significant reduction in unplanned intubation, 1.4% in 2016 vs. 0.7% in 2017.

**Conclusions:**

The standardization of perioperative pulmonary care bundle, including smoking cessation, perioperative pulmonary interventions and early mobilization, significantly reduces the incidence of postoperative pulmonary complications in cancer patients. This technique's implementation was feasible easily even with limited resources in a developing country like Jordan.

## Background

Postoperative pulmonary complications are a leading cause of postoperative morbidity and mortality, increasing critical care and hospital length of stay [1]. Patients undergoing major surgery and who are exposed to general anesthesia have a higher risk for development of postoperative pulmonary complications [2].

According to the American College of Surgeons (ACS) national surgical quality improvement program’s (NSQIP) definition for postoperative pulmonary complications, they are divided into four types; postoperative chest infection (pneumonia), pulmonary embolism ,unplanned reintubation, and using ventilator for > 48 hrs within 30 days postoperatively [3].

Risk factors for postoperative pulmonary complications are categorized by NSQIP into two categories; Patient-Related Factors and Surgery-Related Factors (Table 1). These risk factors were initially taken in consideration in the preoperative assessment. However, NSQIP clearly states that there are certain patient-and surgery-related factors that are *not* associated with an increased risk of postoperative pulmonary complications.

**Table 1:** Risk factors for PPCs by ACS NSQIP

Risk Factors for PPC	
**Patient-Related Factors**	
Age > 60 Years	
Chronic Obstructive Pulmonary Disorder	
American Society of Anesthesiologists (ASA) Class II Or Greater	
Functional Dependence^a^	
Congestive Heart Failure	
Obstructive Sleep Apnea	
Current Cigarette Use	
Impaired Sensorium^b^	
Pulmonary Hypertension	
High-Complexity Operation (Work-Relative Value Units [Rvu] >17)	
Preoperative Sepsis	
Serum Albumin <3.5mg/Dl	
Hypernatremia (Serum Sodium >145)	
**Surgery-Related Factors**	
Prolonged Operation >3 Hours	
Surgical Site^c^	
Emergency Operation	
General Anesthesia	
Perioperative Transfusion	
Residual Neuromuscular Blockade After An Operation	

^a^Partial or total dependence, where partial dependence is defined as the need for devices, equipment, or assistance from another person for some activities of daily living, and total dependence is defined as the inability to perform any activities of daily living without assistance

^b^Acutely confused or delirious patient who is able to respond to verbal or mild tactile stimulation, or mental status changes/delirium in the context of current illness

^c^Highest risk procedures: neurosurgical, head and neck, aortic, thoracic, and upper abdominal

An audit showed an incidence of 5.3% of pulmonary complications postoperatively in major-surgery patients during 2016 at King Hussein Cancer Center, a tertiary cancer center. A multidisciplinary team developed a strategy to reduce postoperative pulmonary complicationsthrough comprehensive patient education and a set of standardized electronic physician orders to specify early postoperative mobilization and pulmonary care.

## Methods

King Hussein Cancer Center is a major provider of surgical oncology services in Jordan and the Middle East. In the year of 2016, we developed and introduced perioperative pulmonary care bundle through a quality improvement methodology that was registered and approved by the hospital’s institutional review board (IRB). We wanted to compare the rate of postoperative pulmonary complications before and after applying the aforementioned bundle.

The study had two stages; (a) we retrospectively analysed data from patients who underwent major oncological surgeries from 1/January/2016 till 31/December/2016 while perioperative pulmonary care bundle was non-mandatory to all cases and recorded the incidence of postoperative pulmonary complications, (b) we analysed data from patients who underwent similar procedures from 1/January/2017 till 31/December/2017 while perioperative pulmonary care bundle was applied to every major case followed by recording the incidence of postoperative pulmonary complications, then those results were compared according to NSQIP criteria.

Patients included in our study were 18 years of age or older and they were scheduled for major cancer surgeries, excluding pregnant women, patients with impaired decisional capacity including neurosurgery patients and those who are planned for tracheostomy or other minor procedures.

After the initial presentation to King Hussein Cancer Center, all patients who are active smokers are referred to the smoking cessation clinic for education and planning to decrease and eventually stop cigarette consumption. Upon admission, one day prior to the surgery, the protocol for eligible patients was explained in details with a voluntary participation. If the patient agreed to participate, a consent form was signed followed by a training session, provided by a respiratory therapist, about aspiration prevention, incentive spirometry use, and chest physiotherapy as part of the perioperative pulmonary care bundle (Appendix 1).

After discharge, the surgical clinical reviewer contacted (either in clinic or via phone) patients on day 10, 20 and 31 to cover the study period proposed. The calls would gather information on surgery related events that happened during that period, and whether readmission or a visit to the emergency department was necessary. On the last call (30 days postoperatively), the patient would be thanked and informed that no further calls will be conducted.

We used SAS® Software version 9.4 (SAS Institute Inc., Cary, NC) for descriptive statistical analysis, using counts and percentages to represent the incidence of Postoperative pulmonary complications. Odds ratio was used to assess the risks after applying the perioperative pulmonary care bundle.

Categorical data were reported as counts and percentages, whereas continuous data were reported as mean ± standard deviation or median (range).

Comparisons between the years and categorical disease characteristics were held using chi-square test or fisher exact test as appropriate, and differences in continuous variables were tested with a student t-test or non-parametric test (Wilcoxon Signed Rank Test).

Multivariate analysis was done using logistic regression model. Adjusted odds ratio and their corresponding 95% confidence intervals were reported.

## Results

During the study period, 1665 patients underwent major surgeries with different specialties (oncological surgeries: general, gynaecology, orthopaedics, head and neck, plastic, thoracic, urology and vascular). 764 (45.9%) patients were from the pre-implementation group in 2016 and 901(54.1%) patients received perioperative pulmonary care bundle in 2017.

Demographically, 890 (53.5%) were males and 775 (46.5%) were females. The mean ± SD age of the group was 53.16 ± 15.28 years, mean body mass index (BMI) was 28.57 ± 6.066 kg/m^2^, mean white blood cells count was 8.17 ± 5.1010. The American Society of Anaesthesiologists (ASA) score for 1662 (99.8%) patients was ≥ 2. A total of 24 patients (1.4%) had chronic obstructive pulmonary disease, and 546 (32.8%) were current smokers.

The most frequent specialty was oncological general surgery (741, 44.5) followed by urology (216, 13.0%) then thoracic (214, 12.9%). The least frequent procedures were plastics and vascular (53, 3.2%) and (3, 0.2%), respectively.

The demographics and risk factors were comparable between the two groups as shown in (Table 2). Patients in the perioperative pulmonary care bundle group had a significant reduction in pneumonia (*p* < 0.004) (Table 3).

**Table 2:** Patient Demographics and Risk Factors

Name	Value	Total	Control Group	PPCB Group	*P*-value
Sex	Female	775	357 (46.7%)	418 (46.4%)	0.891
	Male	890	407 (53.3%)	483 (53.6%)	
Elective Surgery Status	No	30	13 (1.7%)	17 (1.9%)	0.590
	Unknown	20	7 (0.9%)	13 (1.4%)	
	Yes	1615	744 (97.4%)	871 (96.7%)	
Anesthesia Technique	General	1663	764 (100%)	899 (99.8%)	1.000
	Local	1		1 (0.1%)	
	Spinal	1		1 (0.1%)	
Surgical Subspecialty	General Surgery	741	353 (46.2%)	388 (43.1%)	0.462
	Gynecology	185	78 (10.2%)	107 (11.9%)	
	Orthopedics	194	85 (11.1%)	109 (12.1%)	
	Otolaryngology (ENT)	59	26 (3.4%)	33 (3.7%)	
	Plastics	53	31 (4.1%)	22 (2.4%)	
	Thoracic	214	92 (12.0%)	122 (13.5%)	
	Urology	216	98 (12.8%)	118 (13.1%)	
	Vascular	3	1 (0.1%)	2 (0.2%)	
Diabetes	Insulin	125	70 (9.2%)	55 (6.1%)	0.058
	No	1297	587 (76.8%)	710 (78.8%)	
	Non-Insulin	243	107 (14.0%)	136 (15.1%)	
Smoker	No	1119	532 (69.6%)	587 (65.1%)	0.052
	Yes	546	232 (30.4%)	314 (34.9%)	
Dyspnea	At Rest	79	21 (2.7%)	58 (6.4%)	0.001
	Moderate Exertion	40	7 (0.9%)	33 (3.7%)	
	No	1546	736 (96.3%)	810 (89.9%)	
Functional Status Prior to Surgery	Independent	1444	692 (90.6%)	752 (83.5%)	0.001
	Partially Dependent	159	53 (6.9%)	106 (11.8%)	
	Totally Dependent	58	16 (2.1%)	42 (4.7%)	
	Unknown	4	3 (0.4%)	1 (0.1%)	
Ventilator	No	1659	762 (99.7%)	897 (99.6%)	0.693
	Yes	6	2 (0.3%)	4 (0.4%)	
COPD	No	1641	753 (98.6%)	888 (98.6%)	0.996
	Yes	24	11 (1.4%)	13 (1.4%)	
Ascites	No	1641	758 (99.2%)	883 (98.0%)	0.039
	Yes	24	6 (0.8%)	18 (2.0%)	
Cardiac Heart Failure	No	1659	760 (99.5%)	899 (99.8%)	0.422
	Yes	6	4 (0.5%)	2 (0.2%)	
Hypertension	No	1095	492 (64.4%)	603 (66.9%)	0.279
	Yes	570	272 (35.6%)	298 (33.1%)	
Steroid	No	1496	688 (90.1%)	808 (89.7%)	0.801
	Yes	169	76 (9.9%)	93 (10.3%)	
Weight loss > 10%	No	1464	669 (87.6%)	795 (88.2%)	0.676
	Yes	201	95 (12.4%)	106 (11.8%)	
Transfusion	No	1630	753 (98.6%)	877 (97.3%)	0.083
	Yes	35	11 (1.4%)	24 (2.7%)	
Sepsis	None	1560	719 (94.1%)	841 (93.3%)	0.565
	SIRS	76	30 (3.9%)	46 (5.1%)	
	Sepsis	24	13 (1.7%)	11 (1.2%)	
	Septic shock	5	2 (0.3%)	3 (0.3%)	
Emergent Case	No	1644	751(98.3%)	893(99.1%)	0.138
	Yes	21	13 (1.7%)	8 (0.9%)	
Wound Classification	Clean	716	356(46.6%)	360(40.0%)	0.027
	Clean/ Contaminated	868	370(48.4%)	498(55.3%)	
	Contaminated	64	32 (4.2%)	32 (3.6%)	
	Dirty/Infect	17	6 (0.8%)	11 (1.2%)	
ASA Class	ASA 1 - No Disturb	2	1 (0.1%)	1 (0.1%)	0.001
	ASA 2 - Mild Disturb	1392	659(86.3%)	733(81.4%)	
	ASA 3 - Severe Disturb	250	103(13.5%)	147(16.3%)	
	ASA 4 - Life Threat	20	1 (0.1%)	19 (2.1%)	
	None assigned	1		1 (0.1%)	
Age	Mean (95% CI)	53.16 (52.0,54.4)	53.5 (52.6,54.4)	55.0 (18.1,93.7)	0.296
	Median(Min,Max)	54.24 (18.09,93.67)	52.8 (52.0,53.7)	53.7 (18.2,90.4)	
BMI	Mean (95% CI)	28.57 (28.2,29.0)	28.6 (28.2,29.0)	28.0 (13.7,68.9)	0.959
	Median(Min,Max)	28.08 (13.22,68.89)	28.6 (28.2,28.9)	28.1 (13.2,58.9)	
WBC	Mean (95% CI)	8.17 (7.9,8.6)	8.3 (7.9, 8.6)	7.4 (0.7, 136)	0.739
	Median (Min,Max)	7.4 (0.3,136.2)	8.1 (7.9, 8.3)	7.5 (0.3,50.0)

**Table 3:** Outcomes

name	value	Total (*N* = 1665)	Control Group	PPCB Group	*P*-value
Postop Pneumonia	No	1636	743(97.3%)	893(99.1%)	0.004
	Yes	29	21 (2.7%)	8 (0.9%)	
Postop Unplanned Intubation	No	1648	753(98.6%)	895(99.3%)	0.118
	Yes	17	11 (1.4%)	6 (0.7%)	
Postop Pulmonary Embolism	No	1658	761(99.6%)	897(99.6%)	1.000
	Yes	7	3 (0.4%)	4 (0.4%)	
Postop On Ventilator > 48 hrs	No	1656	758(99.2%)	898(99.7%)	0.316
	Yes	9	6 (0.8%)	3 (0.3%)	

Other postoperative pulmonary complications (pulmonary embolism, and on ventilator > 48 hrs) were not affected by the implementation of perioperative pulmonary care bundle (*p* = 1.000, *p* = 0.316, respectively). Even though unplanned intubation did not have a significant difference statistically (*p* = 0.118) but we noticed a decrease in the number of patients after application of the bundle (from 11 (1.4%) to 6 (0.7%)) (Table 3).

## Discussion

A total of 1665 patients were divided into two groups, the control group was 764 participants who underwent cancer surgery in the year of 2016 without adherence to the peri-operative pulmonary care bundle, and the study group was 901 participants who had cancer related surgery in 2017, all of which had the pulmonary care bundle. A comparative descriptive analysis showed a decreased in overall post-operative pneumonia.

The ICOUGH program was developed by Boston University Medical Center in 2012 that was an abbreviation for; incentive spirometry, coughing/deep breathing, oral care, getting out of bed, and head bed elevation. They were able to reduce post-operative pneumonia from 2.13 (observed/expected ratio) to 1.58 (odds ratio) [1]. A modified European protocol was later developed by Manchester University Hospital in the year of 2017 that added to ICOUGH program a pre-operative management plan that included smoking cessation, reduction of alcohol intake, and dietary, and life-style recommendations. Intra-operatively, they recommended close observation for temperature, fluid management, and cardiac monitoring. Post-operatively, they emphasized the importance of pain control, physiotherapy, early nutrition, and chest care/physiotherapy [3].

We designed the perioperative pulmonary care bundle on multiple stages starting with patient education, he/she was instructed to avoid smoking before surgery and referred all active smokers to the smoking cessation clinic to help them quit [4]. On admission, a focus on optimal lung expansion was achieved by chest physiotherapy, deep breathing exercises, incentive spirometry [5, 6]. The interventions are more effective if patient education begins before surgery [7]. Pain control is crucial to achieve more efficient lung exercises [8]. Aspiration prevention is achieved by careful intubation and anesthesia, head elevation after surgery, eating out of the bed and patient-staff education [9]. Post-operative early mobilization facilitates deep breathing, increases lung volumes, and decreases the potential for postoperative pulmonary complications. In a small trial (*n* = 116) of patients undergoing surgery for gastrointestinal cancer, outcomes were evaluated before and after a protocol that included a structured mobilization program by trained nursing staff and walking supervised by a physical therapist beginning on the first postoperative day that resulted in a decrease of postoperative pulmonary complications among those who received early mobilization (OR 0.38, 95% CI 0.12–1.20) [10, 11].

The control group in which the data was collected retrospectively in 2016 might subject our study to a recall bias which we tried to eliminate by assigning the same surgical clinical reviewer to follow-up with each patient in both groups.

Improving cancer patients’ quality of life is considered one of our major goals at.

King Hussein Cancer Center and we are always redefining standards of care based on well-validated protocols. We believe that the success that we were able to achieve with implementation of perioperative pulmonary care bundle should be sustained through continuous education sessions for staff and patients, along with frequent assessment of adequate execution.

Developing countries’ health workers face numerous challenges in patient care due to lack of resources and many other limitations, so establishment of such an easily applicable patient’s care bundles will significantly improve the post-operative outcomes.

## Conclusions

Standardization of perioperative pulmonary care bundle including smoking cessation, perioperative pulmonary interventions, prevention of aspiration and early mobilization significantly reduce the incidence of postoperative pulmonary complications and based on our results, King Hussein Cancer Center implemented a mandatory perioperative pulmonary care bundle as a standard of care to all patients undergoing major surgery. This technique implementation was feasible easily even with limited resources in a developing country like Jordan.

## Data Availability

The Excel sheet for data used to support the findings of this study is available and a copy was attached to the submitted file.

## Appendix

The respiratory therapist educated patients on aspiration prevention, incentive spirometry, chest physiotherapy by adhering to the following:

- Start CPT as soon as possible
- Elevate head of bed at > 45º
- Eat while sitting, do not eat alone if possible
- Keep incentive spirometer within reach
- Incentive spirometry 10 times every hour (3-5 efforts each set) while awake until discharge
- Encourage patient to cough and deep breathe every 2 h
- Ambulate at least once on the day of the operation unless patient arrives from post-anaesthesia care unit late in evening
- Ambulate at least 3 times/day, preferably at mealtimes, with assistance as needed

## Abbreviations

NSQIP: National Surgical Quality Improvement Program
BMI: Body mass index
ACS: American College of Surgeons
IRB: Institutional review board
ASA: American society of anaesthesiologists
